# Operational Efficiency of an Immunization Clinic Attached to Rural Health Training Centre in Delhi, India: A Time and Motion Study

**DOI:** 10.1155/2014/671963

**Published:** 2014-11-06

**Authors:** Varun Kumar, Abha Mangal, Sanjeet Panesar, Geeta Yadav, Richa Talwar, Deepak Raut, Saudan Singh

**Affiliations:** Department of Community Medicine, Vardhman Mahavir Medical College and Safdarjung Hospital, New Delhi 110029, India

## Abstract

*Background*. Obtaining baseline data about current patterns of work is important for assessing the effects of interventions designed to improve care delivery. Time and motion studies allow for the most accurate measurement of structured components. Therefore, the present study was conducted to study the operational efficiency of an immunization clinic in Delhi, India. *Methods*. An observational cross-sectional study was conducted at the immunization clinic of Rural Health Training Centre in Delhi, India, from January 2014 to March 2014. The study composed two stage evaluations, a passive observation and a time and motion study. Systemic random sampling method was used to select 863 mothers/caregivers attending the immunization clinic. *Results*. At the immunization clinic, the study participants spent 64.1% of their total time in waiting. For new cases, the mean time taken for initial registration and receiving postvaccination advice was found to be significantly longer than old cases. Delivering health care services took more time during Mondays and also during the first hour of the day. *Conclusion*. Results of this study will guide public health decision-makers at all government levels in planning and implementation of immunization programs in developing countries.

## 1. Introduction

The quality of available healthcare has been a pertinent issue and is considered as a major hurdle in achieving universal health coverage especially in developing countries. Quality improvements to healthcare can be directly correlated with low morbidity and mortality rates. Improvement in healthcare decreases medical errors and healthcare associated infections. In recent years, healthcare enterprises all over the world have begun applying time and motion studies and system analysis tools to improve their operations and overall efficiency [1].

Obtaining baseline data about current patterns of work is important for assessing the effects of interventions designed to improve care delivery. Time and motion study attempts to track the time records of activities of individuals or group of people. It captures time spent on an activity and thus makes it possible for one to determine how much time is needed to execute that activity and whether time is used efficiently [2]. Time and motion study is a business efficiency technique and as challenges faced healthcare institutions are becoming more and more complex, concepts and disciplines honed in finance, logistics and management are also increasingly being applied to solve healthcare problems [3].

Immunization is considered as one of the most cost effective public health intervention which directly or indirectly prevents the bulk of mortalities in under-five children. Since the Millennium Summit in 2000, immunization has moved centre stage as one of the driving forces behind efforts to meet the Millennium Development Goals (MDGs)—in particular, the goal to reduce deaths among children under five years old (MDG 4). In 2005, the World Health Organization (WHO) and the United Nations Children's Fund (UNICEF) published the Global Immunization Vision and Strategy (GIVS) for the decade 2006–2015 [4].

In spite of continued efforts and millions of dollars poured into Universal Immunization programme (UIP), immunization coverage in India has shown only marginal improvement over last few decades. The data from National Family Health Survey-1 (NFHS-1) shows that only 36% of children were fully vaccinated, but there was very little improvement in NFHS-2 (42%) and NFHS-3 (44%) surveys [5].

Indian Public Health Standards (IPHS), 2012, has laid down certain principles for primary health centres to carry out immunization activities. This includes giving full immunization to all infants and children against vaccine preventable diseases as per guidelines of Government of India (GOI), Vitamin A prophylaxis as per national guidelines, nutritional assessment of infants/children along with plotting of growth chart, health education to mother/caregiver followed by post vaccination advice and maintaining a vaccine record [6].

Although there are many ways to perform any task, one method will be superior to other and the superior method can be determined by observing the time taken to carry out different parts of an activity. Operational efficiency in healthcare refers to proper utilization of resources which can be determined by time and motion studies. Although, time and motion studies are time-consuming they allow for the most accurate measurement of structured components. Since the tasks carried out in immunization clinics are already structured and defined, this design is ideal for evaluating its efficiency [7]. Therefore, the present study was conducted to study the operational efficiency and also to find the time required for various activities at different service points in the immunization clinic attached to a rural health training centre in Delhi, India.

## 2. Materials and Methods

### 2.1. Study Design

An observational cross sectional study was conducted at the immunization clinic of Rural Health Training Centre (RHTC), Najafgarh, which is a field practice area of the Department of Community Medicine, Vardhman Mahavir Medical College and Safdarjung Hospital, New Delhi. The study was done between January and March 2014. The study composed two stage evaluation, a passive observation and a time and motion study.

### 2.2. Study Participants

The study participants included mothers/caregivers attending immunization clinic with their children. The registration records present at the immunization clinic for the last two years were reviewed and the average daily registration including both the old and new cases were found to be 70 in number. Every fifth mother/caregiver registering in the immunization clinic on the day of study was selected by systemic random sampling method. Considering the average number of working days in a month to be 20, the minimum sample size to be achieved was fixed at 840. Finally, 863 study participants were included in the study. Informed written consent was obtained from each mother/caregiver included in the study.

### 2.3. Data Collection 

#### 2.3.1. Stage 1 (Passive Observation)

We spent one week initially passively observing the immunization clinic to become familiar with the specific core immunization tasks that each staff member is responsible for. At the end of stage 1, we found that the staffs present in the immunization clinic were involved in following core functions.

1. Registration of new and old cases (collecting demographic information like name, sex, date of birth, address, etc.).
2. Nutritional assessment of under-5 children (recording weight, length/height, plotting in growth chart, etc.) and medical history review.
3. Giving nutritional advice and health education about preventing common childhood illnesses.
4. Vaccine administration and record keeping regarding vaccination (vaccine, dosage, dose number, site of vaccination, etc.).
5. Post vaccination advice (regarding common side-effects of vaccine and when to seek medical attention).

#### 2.3.2. Stage 2 (Time and Motion Study)

Predesigned and pretested schedules were used to record time and other information and presynchronized stop watches were used to record total time taken for each of the above mentioned activities. Based on the layout of the immunization centre, we selected an unobtrusive location to observe the staff member close enough to the interaction to see and/or hear it take place to record its duration but we stayed at a great enough distance to not interfere with the process. Start and end times were based on both visual and verbal cues. For example, the “start time” of an observation may have been the initial registration-related task for a new client (i.e., began a new data form, engaged the client in related conversation). “End time” was defined as the point when a staff member had completed all tasks associated with that particular activity.

### 2.4. Data Analysis

Statistical analysis was done using SPSS version 21. The variables used in the analysis were waiting time at different service delivery points, service delivery time at different activity points, time taken for nutritional assessment, nutritional advice, vaccine administration, and postvaccination advice. The time has been expressed as mean and standard deviation. Trimmed mean (5% of values at both extremes were trimmed) was used in certain places as data was very much skewed leading to large standard deviations. The mean observation time per task was calculated and average times were compared using analysis of variances unpaired* t*-test.

## 3. Results 

Out of 863 study subjects, 115 came for the first time to the immunization clinic while 648 were old cases. Each study participant on an average had spent 2614.9 ± 817.1 seconds in immunization clinic. The average total waiting time was 1677.2 ± 585.1 seconds while the average total service delivery time received by each study participant was 937.7 ± 304.1 seconds. The average waiting time at different service delivery points is given in Table 1.

Table 1 shows the waiting time at different service delivery points. The study participants had spent 64.1% of their time at immunization clinic in waiting to get the service. Most of the total waiting time was spent in initial registration (50.3%) followed by health education (19.8%). The waiting time spent for vaccine administration and post vaccination advice were 11.6% and 11.1% of the total waiting time, respectively.

Table 2 shows the service delivery time at different activity points. The mean time taken during initial registration was 181.3 seconds and it constitutes 19.3% of the total activity time. The mean time taken for registering new cases was longer (286.6 ± 211.2 seconds) when compared with the average time taken for registering old cases (146.3 ± 90.8 seconds) and the difference was found to be statistically significant (*P* value = 0.000).

The mean time for nutritional assessment was 155.8 seconds and it took 16.6% of the total activity time. Mothers/caregivers of children spent most of their time in health education and vaccine administration which constituted 21.9% and 25.9% of the activity time, respectively. The mean time spent in postvaccination advice was 152.7 seconds. The time taken for giving post vaccination advice for new cases was longer (284.6 ± 205.1 seconds) when compared with old cases (108.3 ± 106.2 seconds) and the difference was found to be statistically significant (*P* value = 0.000).

When service delivery time was compared with the day of visit, it was found that the maximum time for initial registration was on Monday (204.2 seconds). Tuesday took maximum time for nutritional assessment (168.9 seconds) and postvaccination advice (150.4 seconds). Friday took maximum time for giving health education (211.8 seconds) while Thursday and Monday took almost equal time for administering vaccines (Table 3).

The service delivery time was at its maximum for all the functions of immunization clinic between 10 to 11 AM on any day. This was followed by 11 AM to 12 PM session. The service delivery time was at its minimum between 12 PM to 1 PM (Table 4).

## 4. Discussion

Global Immunization Vision and Strategy (GIVS) for the decade 2006–2015 was published by World Health Organization (WHO) and the United Nations Children's Fund (UNICEF) with an overriding focus on the need to ensure equity in access to vaccines and immunization. The strategy sets out the steps that the immunization community needs to take in order to contribute fully to the attainment of the MDG mortality reduction targets. Implementing the strategy calls for four main approaches: protecting more people; introducing new vaccines and technologies; integrating immunization with other components in the health system context; and immunizing in the context of global interdependence. It has also called for better operational efficiency of already functioning immunization clinics [4].

Many studies have found that long waiting times and insufficient and inefficient staff as major obstacles faced by healthcare facilities in developing countries [8]. In the present study, it was observed that study participants had to spend 64.1% of their time at the immunization clinic in waiting to get service from immunization clinic. About half of their waiting time was spent on initial registration. The long waiting time could be due to lack of adequate health care workers. Though there are four healthcare workers posted in the immunization clinic, it is not proportionate to the amount of cases they handle.

Timeliness of services rendered at the primary health care level which also includes immunization clinic impacts positively upon the perception of quality of services among the clients [9]. In our study, we have observed that the mothers/caregivers spent only one-third of their total time at the immunization clinic in receiving healthcare services. This may negatively affect their perception about receiving immunization at primary health care level.

Different components of work require varying time to complete the task. By finding the time required for individual subcomponents, suitable measures can be explored to complete the task in lesser time. In the present study, most of the time spent by study participants in receiving service was in getting vaccination followed by health education. Further we also found the new cases spent more time at initial registration and in getting postvaccination advice than old cases. This may be due to healthcare workers taking more time to enter various sociodemographic details during initial registration. These results were similar to a study conducted in Kolkata which also shows maximum time (46.3%) at the immunization clinic were spent in getting postvaccination advice [8].

Ensuring access to healthcare equally on all days is important for achieving universal health coverage. In our study, we had found that Mondays took maximum time for initial registration. This may have been due to more number of people coming to immunization clinic on the first day of the week (*n* = 233). Such differential access to health centre can be due to number of staff present on the particular day of study, unequal efficiency of the number of staffs and availability of vaccines uniformly.

The service delivery time was at its maximum in the beginning of the day and gradually decreases during subsequent sessions. Again various factors could be responsible for this difference observed in relation to time of visit like total case load, waiting time and service time in getting various services at the immunization clinic. However, these variations need to be studied in depth.

The study represents one of the very few time and motion studies of functioning of immunization clinic in developing countries, and as such, provides a useful baseline for future studies. By identifying the bottlenecks and constraints in the system, the quality and efficiency of immunization services can be improved. Perhaps, we think that this study will help in the initiation of further in-depth analysis of constraints and bottlenecks in implementation of immunization program in developing countries and in providing guidelines for optimal functioning of the system.

The study also has some limitations in itself being an observational study design. The results of the study cannot be generalized since the data was collected from single immunization clinic attached to a rural health training centre in Delhi, India. The study can be done on a wider area and further research can be done to evaluate the effectiveness of integrating the time and motion model into the existing immunization program and subsequent remedial steps for optimal functioning of the program.

## 5. Conclusion

Efficient functioning of immunization clinics is essential for achieving universal immunization against all vaccine preventable diseases and also in achieving millennium development goals. Management of time at various levels of healthcare system should be recognized so that necessary remedial actions can be initiated for optimal functioning of the healthcare system. Additionally, results of this study will guide public health decision-makers at all government levels in planning and implementing immunization programs and also during other public health interventions.

## Figures and Tables

**Table 1:** Waiting time at different service delivery points (*n* = 863).

Activity	Mean ± SD (in seconds)	% of total waiting time
Initial registration	843.6 ± 701.3	50.3
Nutritional assessment	120.4 ± 95.8	7.2
Health education	332.7 ± 161.4	19.8
Vaccine administration	194.1 ± 86.2	11.6
Postvaccination advice	186.4 ± 134.1	11.1

**Table 2:** Service delivery time at different activity points (*n* = 863).

Activity	Mean ± SD (in seconds)	% of total activity time
Initial registration	181.3 ± 132.3	19.3
Nutritional assessment	155.8 ± 108.6	16.6
Health education	205.5 ± 163.7	21.9
Vaccine administration	242.4 ± 126.4	25.9
Postvaccination advice	152.7 ± 117.3	16.3

**Table 3:** Service delivery time (trimmed mean) in relation to day of visit.

Activity	Time (trimmed mean) in seconds					
	Monday	Tuesday	Wednesday	Thursday	Friday	Saturday
Initial registration	204.2	164.5	178.4	182.3	172.1	184.1
Nutritional assessment	153.2	168.9	157.6	152.4	155.2	150.6
Health education	182.5	209.7	201.3	199.4	211.8	203.9
Vaccine administration	250.1	241.5	245.4	251.6	244.9	239.2
Postvaccination advice	150.4	162.4	155.7	149.8	154.2	152.4

**Table 4:** Service delivery time (trimmed mean) in relation to time of visit.

Activity	Time (trimmed mean) in seconds		
	10-11 AM	11 AM-12 PM	12 PM-1 PM
Initial registration	185.3	178.1	168.7
Nutritional assessment	161.3	154.5	142.4
Health education	219.6	207.4	198.8
Vaccine administration	261.4	247.3	135.1
Postvaccination advice	168.5	154.9	141.2
